# Supplementary material for: A versatile Halo- and SNAP-tagged BMP/TGFβ receptor library for quantification of cell surface ligand binding
Source: Commun Biol. 2023 Jan 12;6:34. doi: 10.1038/s42003-022-04388-4 (PMC9837045; doi:10.1038/s42003-022-04388-4)
Supplement: Supplementary file 2 — Supplementary Information [file 42003_2022_4388_MOESM2_ESM.pdf]

Supplementary Information

A versatile Halo- and SNAP-tagged BMP/TGFβ receptor library for quantification of cell surface ligand binding

Jerome Jatzlau[1,*], Wiktor Burdzinski[1,2,*], Michael Trumpp[1], Leon Obendorf[1], Kilian Roßmann[3], Katharina Ravn[4], Marko Hyvönen[4], Francesca Bottanelli[1], Johannes Broichhagen[3], Petra Knaus[1,2, †]

[1] Institute of Chemistry and Biochemistry - Biochemistry, Berlin, Germany.

[2] Berlin-Brandenburg School for Regenerative Therapies (BSRT), Berlin, Germany.

[3] Leibniz-Forschungsinstitut für Molekulare Pharmakologie, Berlin, Germany.

[4]Department of Biochemistry, University of Cambridge, Cambridge, UK

† Corresponding author

E-Mail: petra.knaus@fu-berlin.de

* These authors contributed equally

This PDF file includes:

Supplemental Figures 1-15

Supplementary Table 1. Oligonucleotides used for cloning.

Synthesis procedure: 2-(7-(Bis(methyl-d_3)amino)-3-(bis(methyl-d_3)iminio)-5,5-dimethyl-3,5-dihydrodibenzo[*b*,*e*]silin-10-yl)-4-(((2,5-dioxopyrrolidin-1-yl)oxy)carbonyl)benzoate (**NHS-SiR-d12**)

Supplemental Figures:

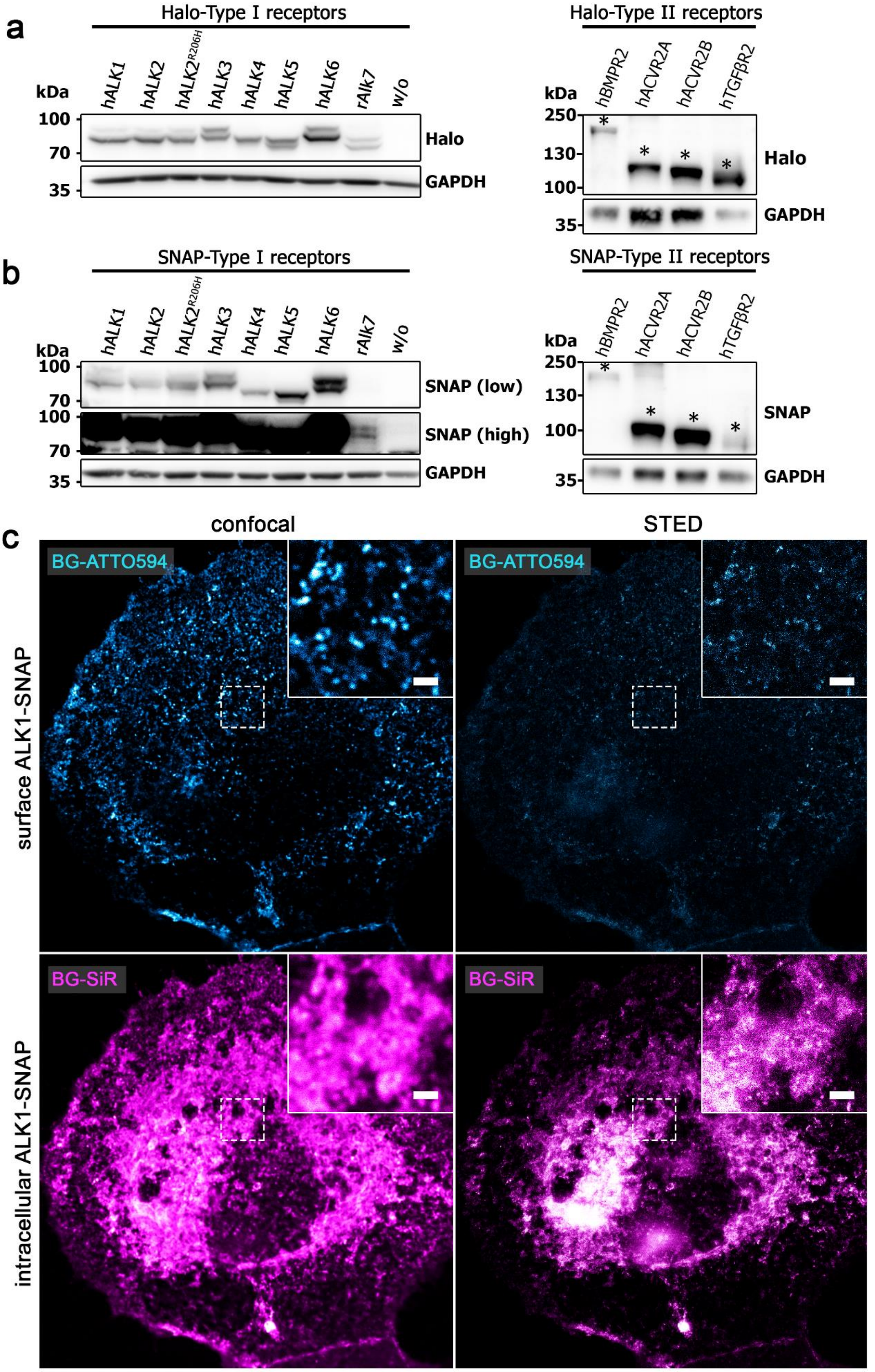

Supplementary Figure 1: ***Supplement to Fig.1*****, a-b** Expression of **a** Halo- and **b** SNAP-tagged receptors in COS-7 cells validated by Western blotting with specific antibodies directed against the Halo- or SNAP-tag, using GAPDH as loading control. **c** Discrimination between surface and cytosolic receptor populations. COS-7 cells transiently expressing ALK1-SNAP were 24 hours post transfection incubated with BG-ATTO594 (impermeable; cyan) followed by BG-SiR (permeable; magenta) incubation allowing for staining of the surface receptor population and the cytosolic receptor population, respectively. Confocal images (left) at a resolution of 60 x 60 nm/pixel compared with STED images (right) at a resolution of 20 x 20 nm/pixel. Scale bar ≙ 1µm.

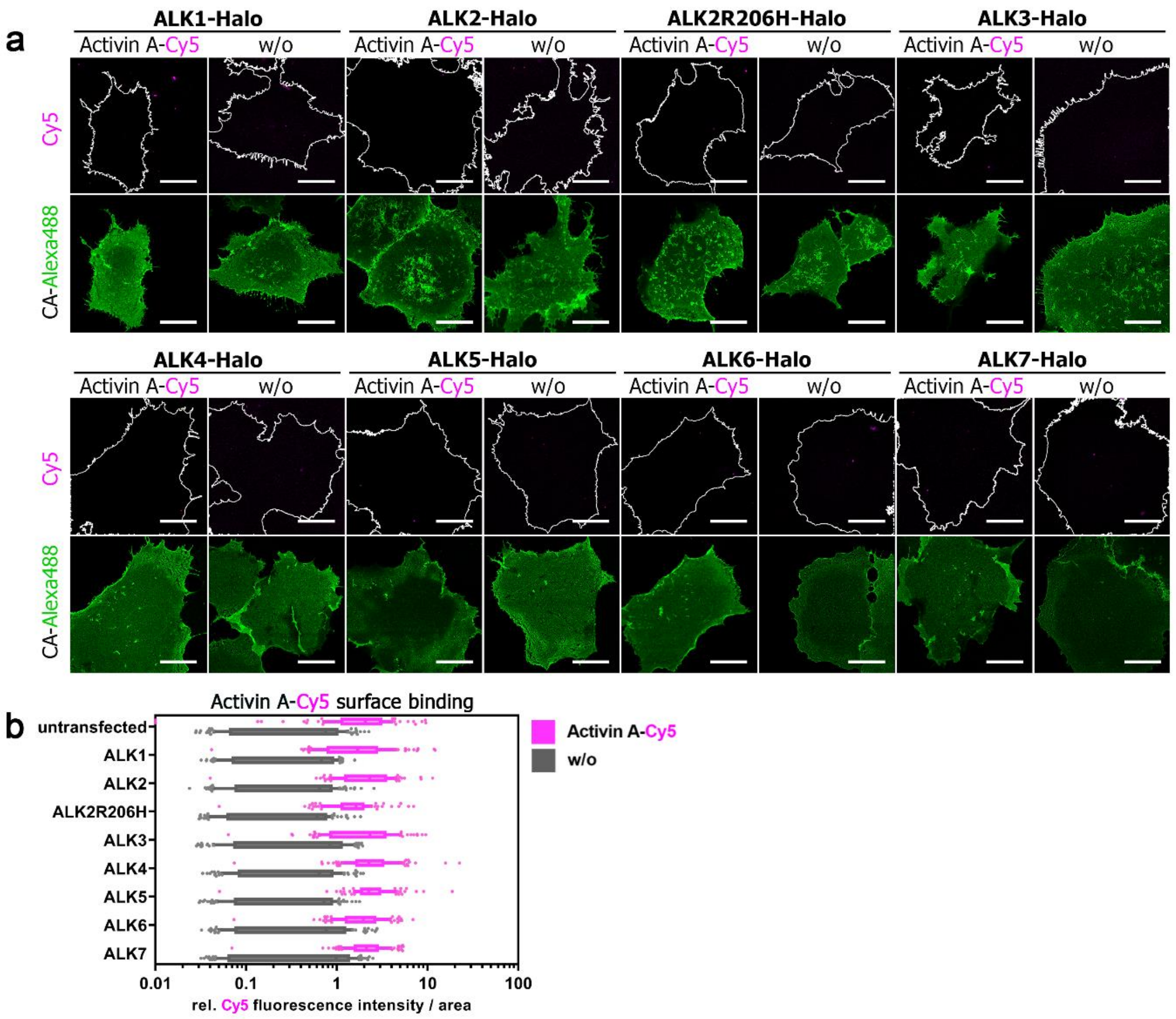

Supplementary Figure 2: ***Supplement to Fig.2,*** **a** COS-7 cells were seeded on coverslips and transfected with indicated Halo-tagged type I receptor constructs. 24 hours post transfection, cells were incubated with fluorescent Halo-tag substrate CA-Alexa488 (green) and Activin-Cy5 (magenta) for 30 minutes at 4 °C, fixated with methanol for 5 minutes at room temperature and mounted on glass slides. Cells were imaged at a confocal microscope and 10 cells per condition and replicate were analyzed with a semi-automated Fiji ImageJ macro pipeline for assessment of fluorescent growth factor binding (Activin A-Cy5 signal intensity) and fluorescence intensity of receptors (CA-Alexa488). Four ROIs of 100 µm^2 were quantified in each cell. **a** Representative confocal microscopy images of COS-7 cells transiently expressing the Halo-tagged type I receptor library incubated with CA-Alexa488 and simultaneously stimulated with Activin A-Cy5 or PBS as control. Scale bar ≙ 20 µm. **b** Activin A-Cy5 surface binding represented as relative fluorescence intensity per area. Data is shown as F.I. ± SD. (n = 3 independent experiments) (CA: chloroalkane).

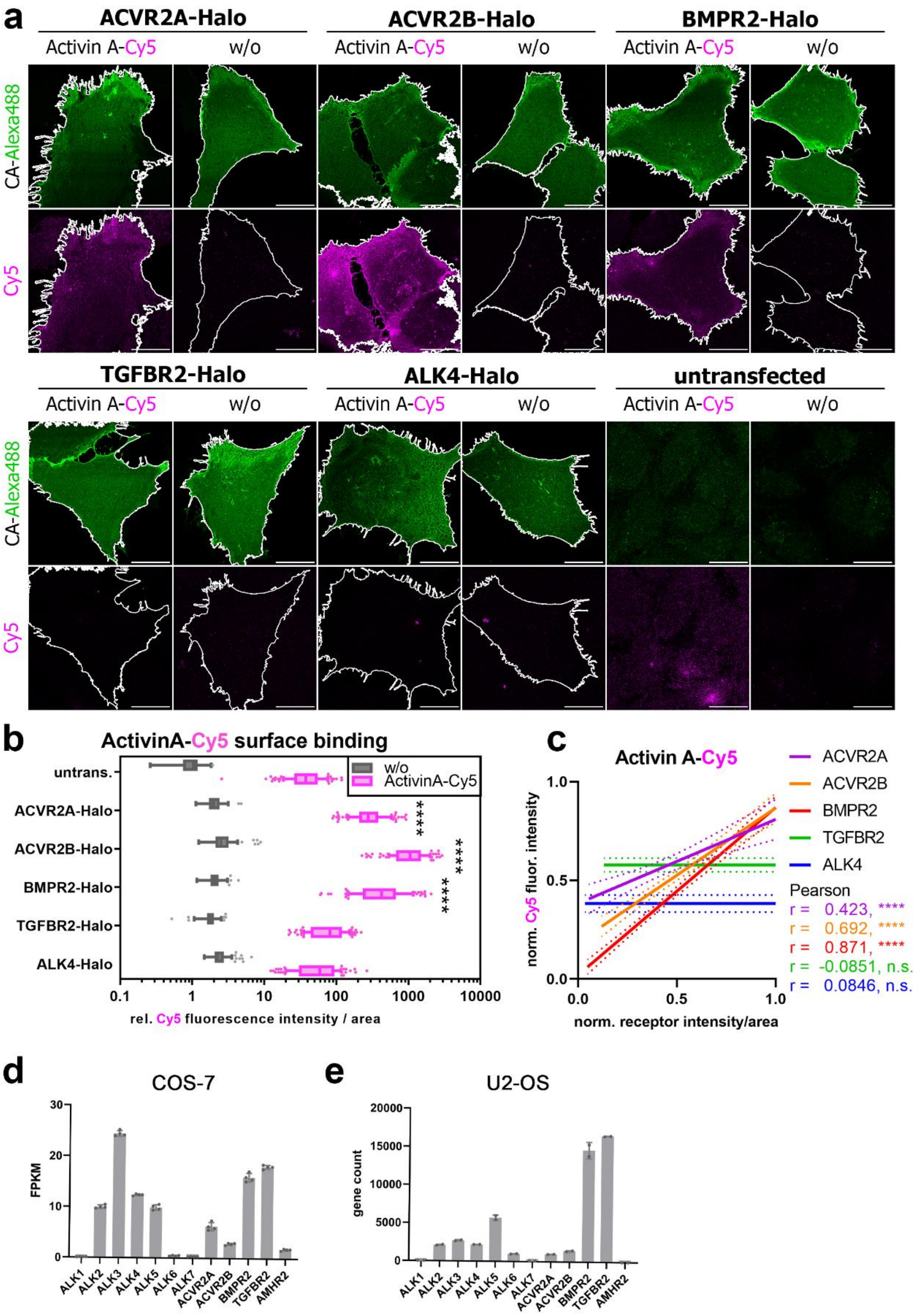

Supplementary Figure 3: ***Supplement to Fig.2*** **a-c** Transiently transfected U2-OS cells expressing ACVR2A-, ACVR2B-, BMPR2-, TGFBR2- or ALK4-Halo were 24 hours post transfection simultaneously incubated with Halo-tag substrate CA-Alexa488 (green) and Activin A-Cy5 (magenta). Data shown is derived from 3 independent experiments. **a** Representative confocal microscopy images of Activin A-

Cy5 stimulated U2-OS cells expressing type II receptors (ACVR2A, ACVR2B, BMPR2, TGFBR2) or type I receptor ALK4. Scale bar ≙ 20 µm. **b** Activin A-Cy5 surface binding represented as fluorescence intensity per area relative to untransfected cells. Data is shown as F.I. ± SD. Significance was calculated using two-way ANOVA and Tukey's post-hoc test. ****$p < 0.0001$ ≡ significant relative to all stimulated conditions. **c** Linear regression and correlation analysis of ligand:receptor binding based on normalized Cy5-fluorescence intensity and normalized receptor fluorescence (CA-Alexa488) per area (n = 3). **d-e** Endogenous BMP & TGFβ receptor expression in COS-7 (GSM5461004) and U2-OS cells (GSM4255919) extracted from publicly available RNA-Seq. data (GEO Data base)[1,2] .

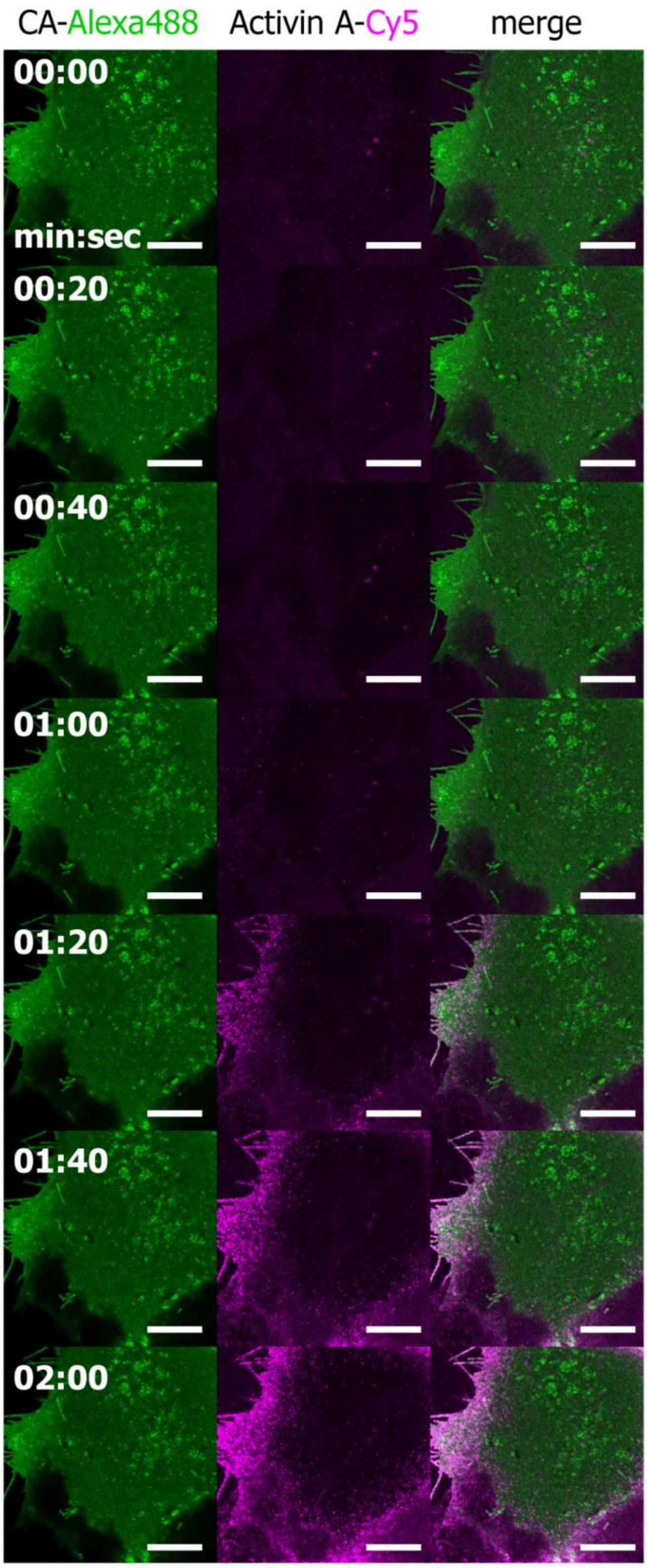

Supplementary Figure 4: ***Supplement to Fig.2,*** Diffusion and binding of Activin A-Cy5 on COS-7 cells in live cell imaging (LCI). COS-7 cells were seeded on 35mm glass bottom dishes (Cellvis) and transiently transfected with ACVR2B-Halo. 24 hours post transfection, cells were stained with CA-Alexa488 for 30 minutes at 4 °C and stored on ice until image acquisition. Stimulation with 2 nM Activin A-Cy5 and imaging was performed in a LCI chamber (okolab) at 37°C. A total of 60 images were acquired over a time of 2 minutes.

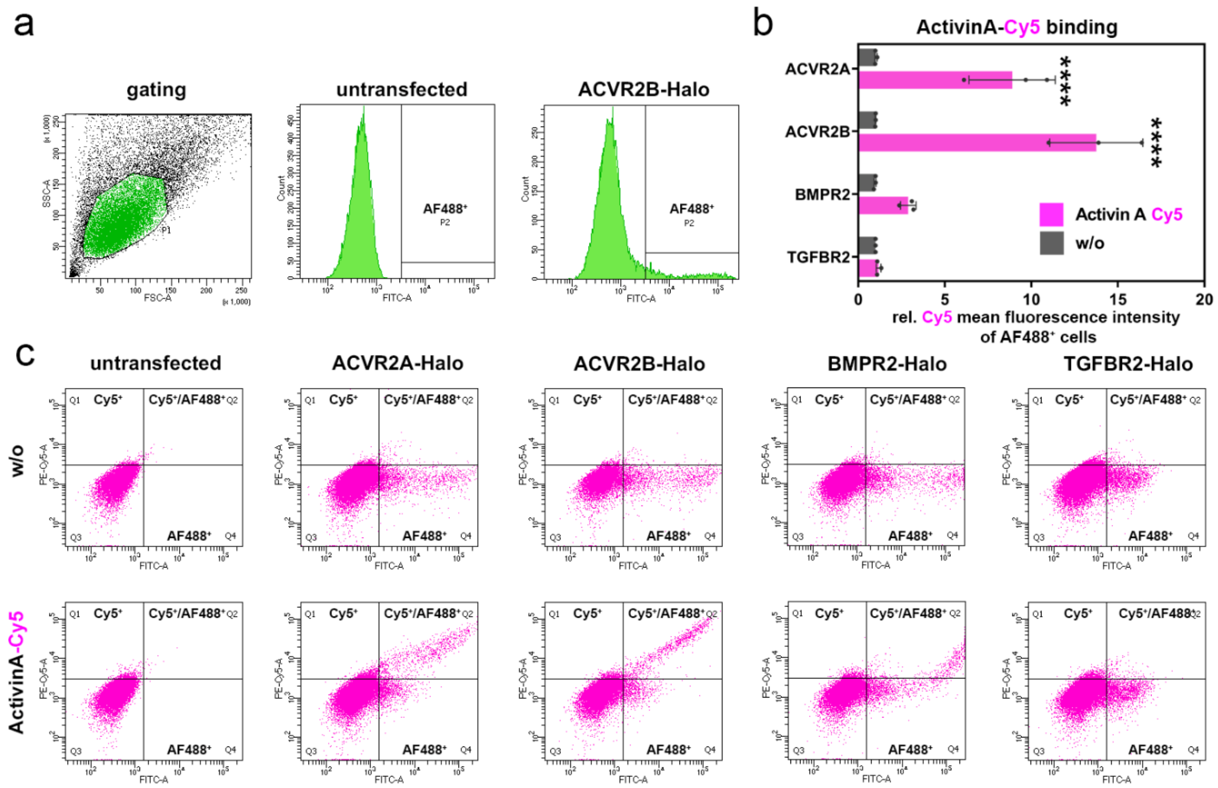

Supplementary Figure 5: ***Supplement to Fig.2,*** **a-c** COS-7 cells were seeded in 6-wells and transfected with indicated Halo-tagged type II receptor constructs. 24 hours post transfection, cells were incubated with fluorescent Halo-tag substrate CA-Alexa488 (green) and Activin-Cy5 (magenta) for 30 minutes at 4 °C and detached with Accutase and analysed using FACS. **a** FACS plots show the side and forward scatter (area), count/FITC-A plot, and the gates used to analyse transfected AF488$^+$ cells only. Transfected cells were gated for 1000 AF488$^+$ cells in gate P2. **b** Activin A-Cy5 surface binding is represented as fluorescence intensity relative to unstimulated (w/o) TGFBR2 control. Data is shown as F.I. ± SD. Significance was calculated using two-way ANOVA and Dunnett's post-hoc test. **** $p < 0.0001$ ≡ significant relative to Activin A-Cy5 stimulated TGFBR2-expressing cells. **c** Plots show AF488$^+$ versus Cy5 fluorescence. Simple gating by quadrants allowed to depict the receptor-expression dependent increase in Activin A-Cy5 binding for ACVR2A, ACVR2B and BMPR2 (Q2), whereas untransfected or TGFBR2 transfected cells show no detectable Activin A binding. Figure is representative of three independent experiments.

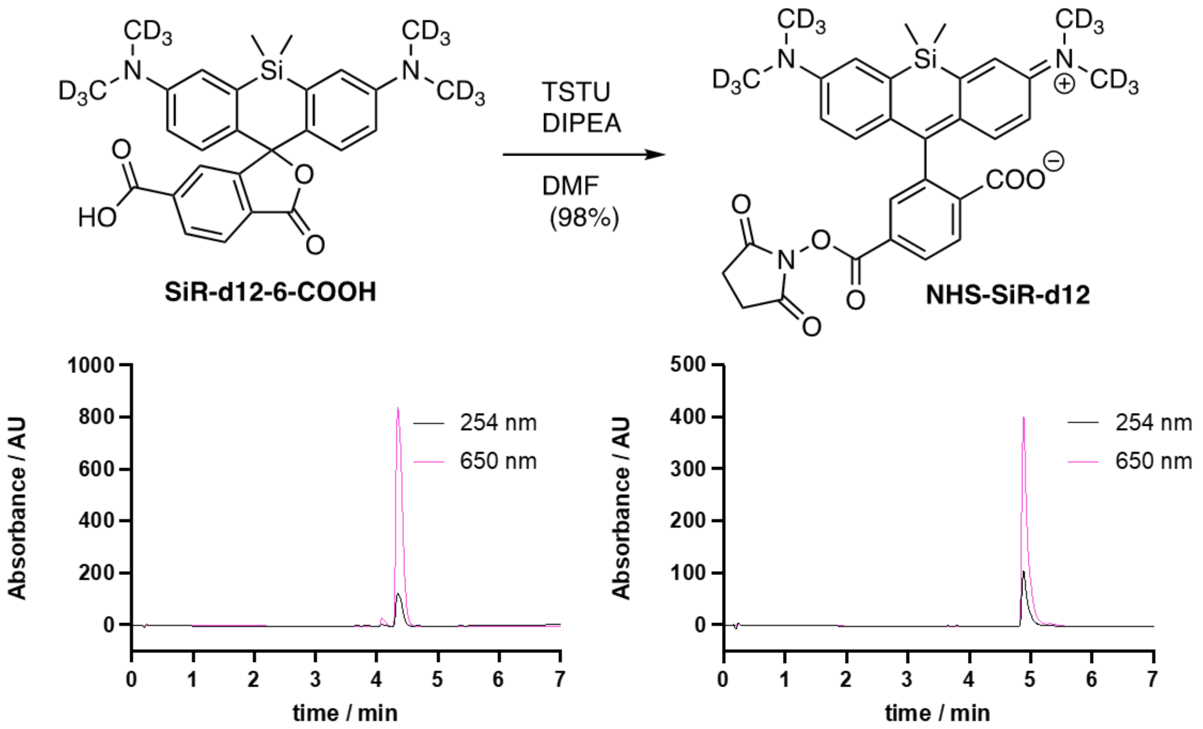

Supplementary Figure 6: ***Supplement to Fig.3,*** Synthesis of NHS-SiR-d12. SiR-d12-6-COOH is activated by means of TSTU in DIPEA and DMF and the corresponding active ester is collected by RP-HPLC. LCMS of both substances below indicates purity.

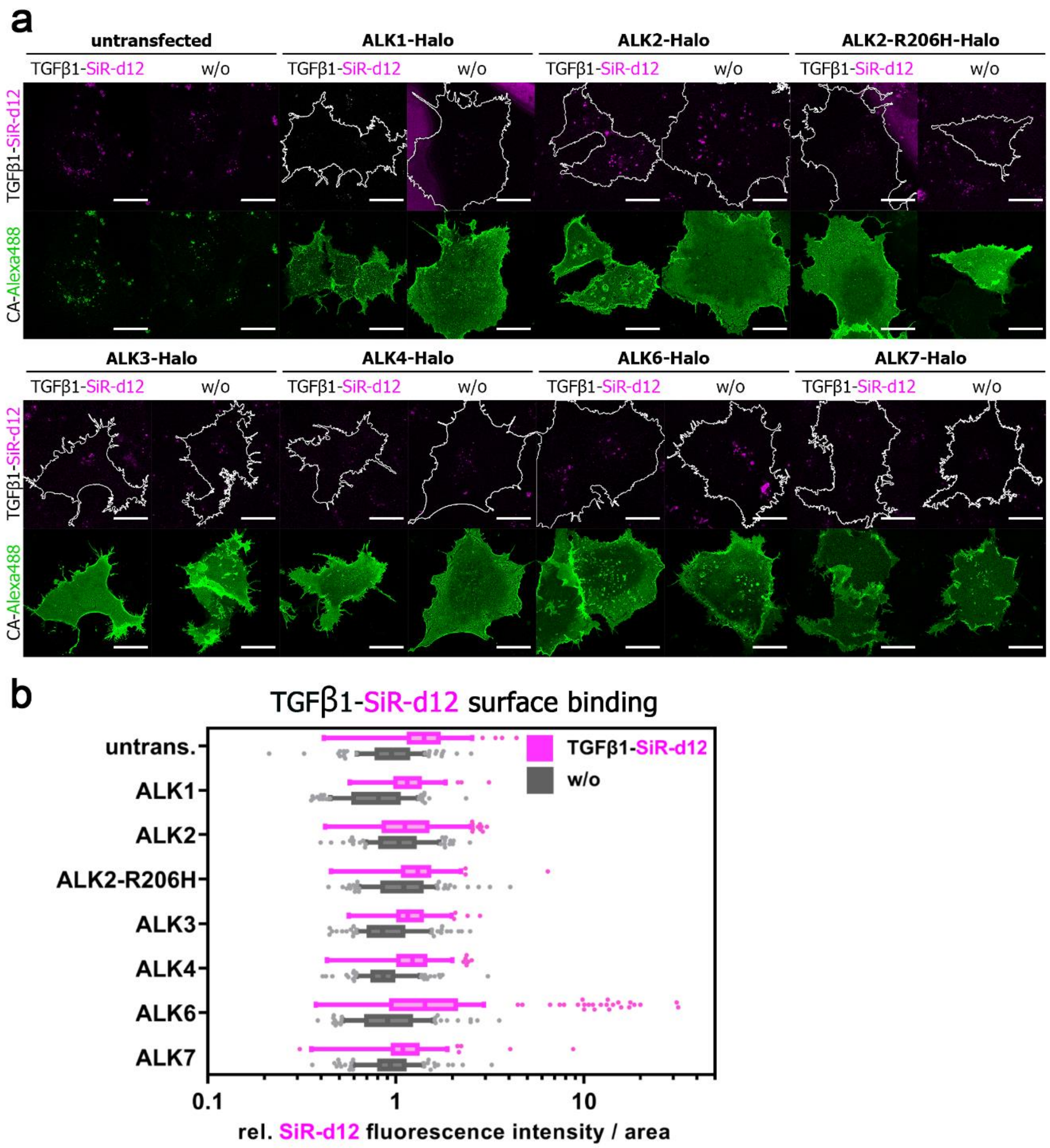

Supplementary Figure 7: ***Supplement to Fig.3,*** Transiently transfected COS-7 cells expressing Halo-tagged type I receptor library were 24 hours post transfection simultaneously incubated with Halo-tag substrate CA-Alexa488 (green) and TGFβ1-SiR-d12 (magenta). **a** Representative confocal microscopy images. Scale bar ≙ 20 µm. **b** TGFβ1-SiR-d12 surface binding represented as relative fluorescence intensity per area. Data is shown as F.I. ± SD. (n = 3 independent experiments).

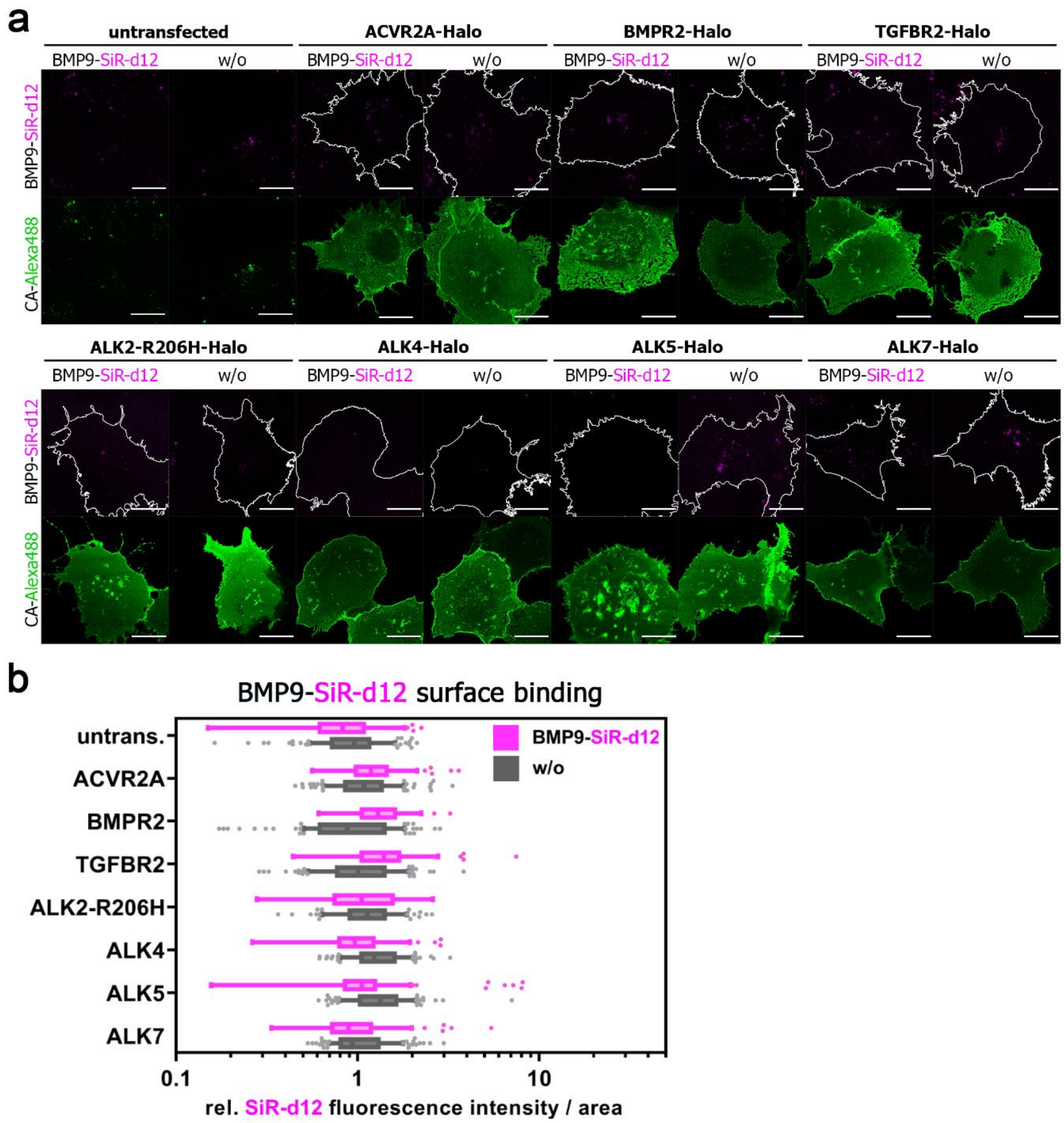

Supplementary Figure 8: ***Supplement to Fig.3,*** Transiently transfected COS-7 cells transiently expressing Halo-tagged type II receptors ACVR2A-Halo, ACVR2B-Halo, BMPR2-Halo or type I receptors ALK2-R206H-Halo, ALK4-Halo, ALK5-Halo, ALK7-Halo were 24 hours post transfection simultaneously incubated with Halo-tag substrate CA-Alexa488 (green) and BMP9-SiR-d12 (magenta). **a** Representative confocal microscopy images. Scale bar ≙ 20 µm. **b** BMP9-SiR-d12 surface binding represented as relative fluorescence intensity per area. Data is shown as F.I. ± SD. (n = 3 independent experiments).

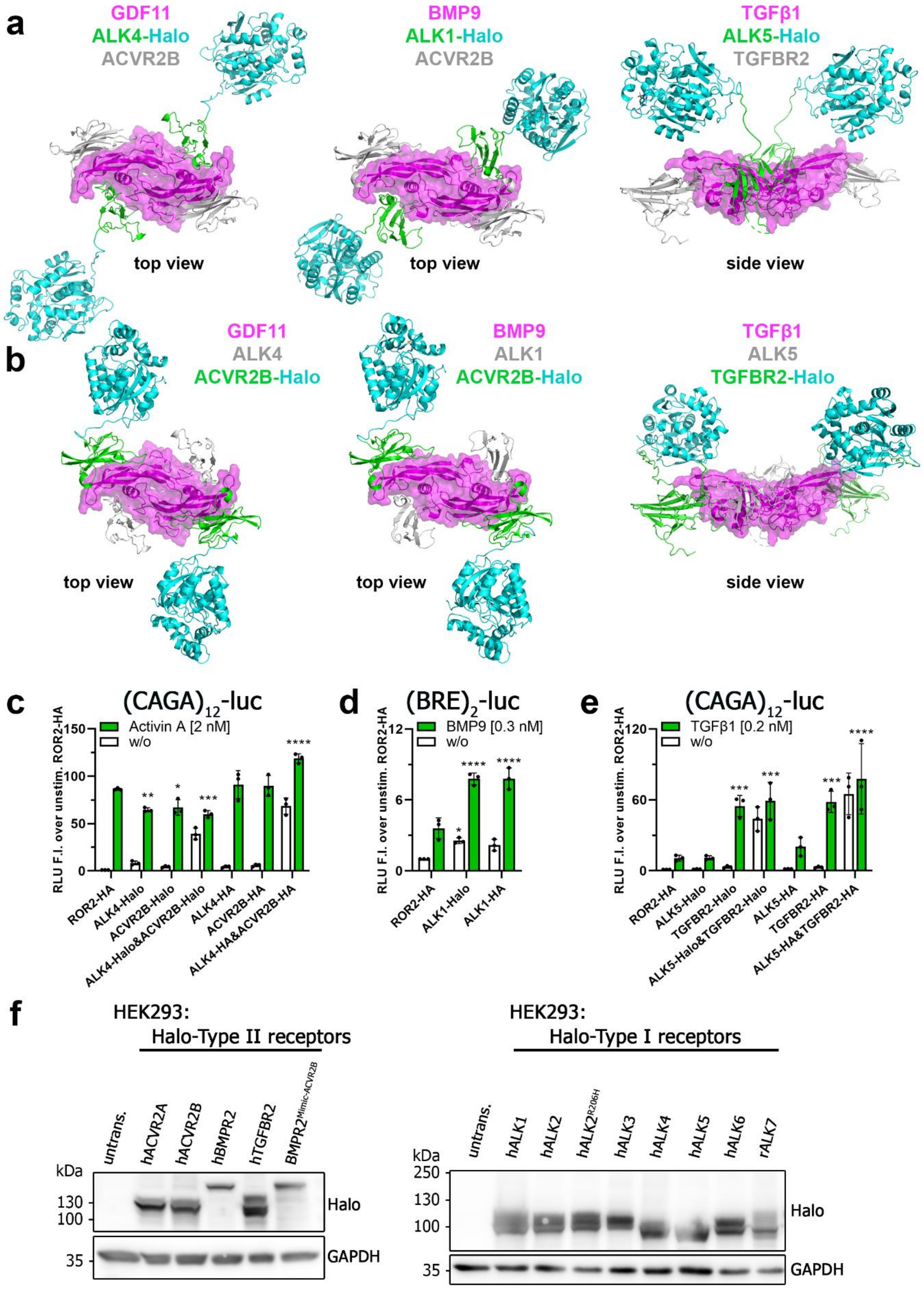

Supplementary Figure 9: ***Supplement to Fig.3*****, a-b** Homology modelling of different Halo-tagged receptor complexes to evaluate the sterically hinderance exerted by the Halo-tag on tetrameric receptor formation. Flexible, long Gly5 linkers allow the formation of tetrameric receptor complex including Halo-tagged receptors. **a** Tagged type I receptors, from left to right: GDF11:ALK4-

Halo:ACVR2B (derived from PDB 7MRZ,[3]) BMP9:ALK1-Halo:ACVR2B (4FAO,[4]) TGFB1:ALK5-Halo:TGFBR2 (derived from PDB 3KFD,[5]). **b** Tagged type II receptors (left to right): GDF11:ALK4:ACVR2B-Halo (derived from PDB 7MRZ,[3]) BMP9:ALK1:ACVR2B-Halo (derived from PDB 4FAO,[4]) TGFB1:ALK5:TGFBR2-Halo (derived from PDB 3KFD,[5]). Halo-fused-receptor models were generated using the PDB entry 5UXZ [6] for the Halo-tag structure and Rosetta Commons modelling suite after preparation in PyMol. **c-e** After one day of transfection with **d** the SMAD1/5/8-sensitive $(BRE)_2$-luciferase reporter or **e** the SMAD2/3-sensitive $(CAGA)_{12}$-luciferase reporter and RLTK-luc in combination with indicated respective **c** Activin A-, **d** BMP9- or **e** TGFβ1 receptors. HEK293t cells were starved for 5 h and stimulated with Activin A (2 nM), BMP9 (0.3 nM) or TGFβ1 (0.2 nM) overnight. Relative Luminescence Units (RLU) are expressed as mean fold induction ±SD over unstimulated ROR2-HA transfected control cells (n=3 independent experiments). Statistical significance relative to unstimulated ROR2-HA was calculated using two-way ANOVA and Tukey's post-hoc test. **f** Expression of Halo-tagged type I and II receptors in HEK cells validated by Western blotting with a specific antibody directed against the Halo-tag, using GAPDH as loading control.

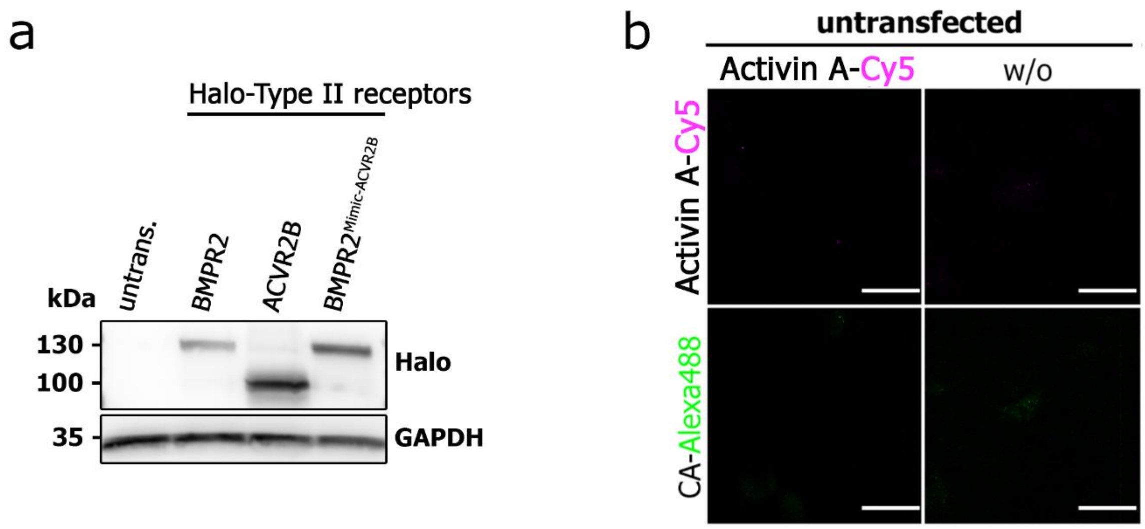

Supplementary Figure 10: ***Supplement to Fig.4*****, a** Expression of Halo-tagged receptors BMPR2-Halo, ACVR2B-Halo and $BMPR2^{Mimic\text{-}ACVR2B}$-Halo validated by Western blotting. Halo-tagged receptors transiently expressed in COS-7 cells validated by Western blotting with specific antibodies directed against Halo-tag, using GAPDH as loading control. **b** Untransfected COS-7 cells were 24 hours post transfection simultaneously incubated with Halo-tag substrate CA-Alexa488 (green) and Activin A-Cy5 (magenta). Representative confocal microscopy images of untransfected COS-7 cells incubated with CA-Alexa488 and simultaneously stimulated with Activin A-Cy5 or PBS as control. Scale bar ≙ 20 µm.

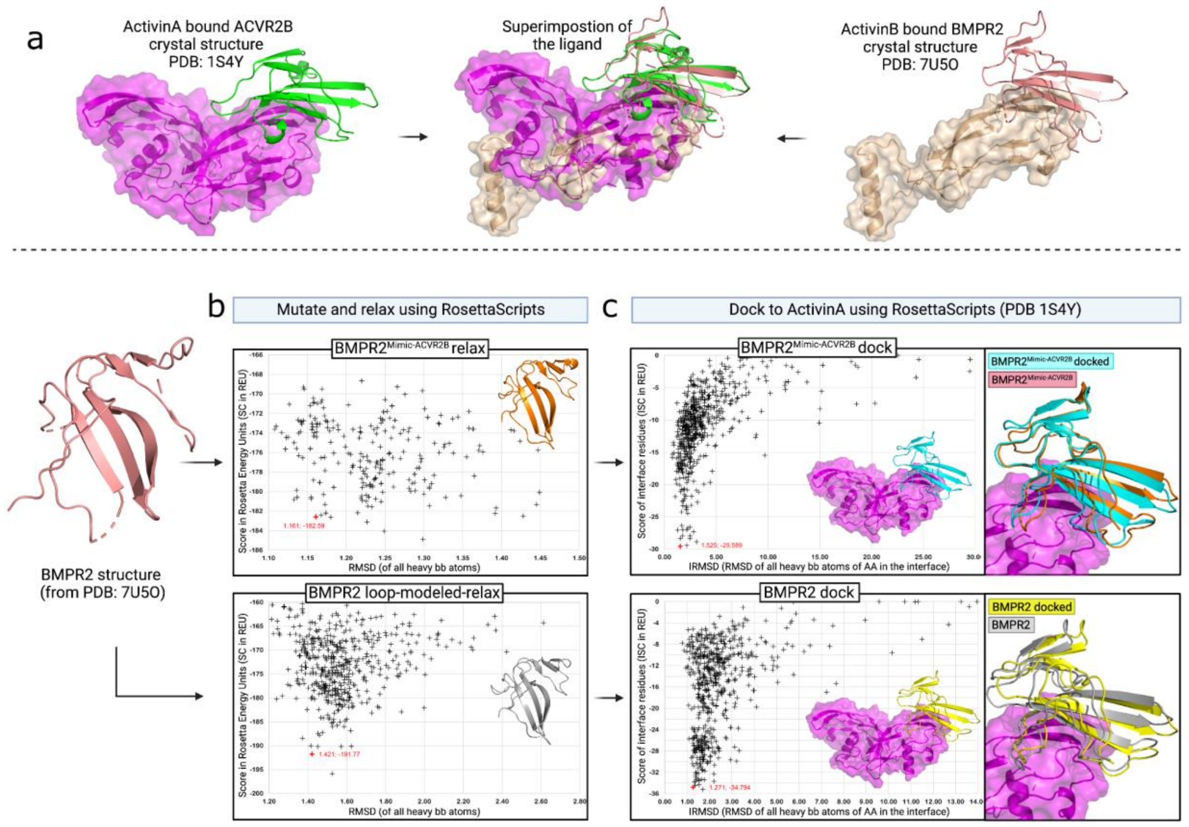

Supplementary Figure 11: ***Supplement to Fig.4*** Simplified, schematic overview of the *in silico* homology modelling of BMPR2$^{\text{Mimic-ACVR2B}}$ and docking of BMPR2 and BMPR2$^{\text{Mimic-ACVR2B}}$ to Activin A using the Rosetta Commons Modelling Suite. **a** Cartoon and surface representation of the crystal structure 1S4Y of ACVR2B bound to Activin A and the crystal structure 7U5O of BMPR2 bound to ActivinB. A superimposition of the ligands (Activin A and Activin B) reveals similar binding epitope. Therefore the input for docking of BMPR2 used this super imposition keeping the chains of BMPR2 and Activin A. **b** RMSD of all heavy backbone atoms vs. the Rosetta-Score (using scoring function ref2015) is plotted for 500 homology modelling attempts of BMPR2$^{\text{Mimic-ACVR2B}}$ (upper, derived from the BMPR2 crystal structure (7U5O) by mutating and relaxing using the Rosetta Commons Modelling suite) and 500 attempts to model missing loops to obtain continuous BMPR2 structure. The Datapoint of the final homology models (cartoon representation in the corner of the plot) is highlighted in red. **c** After superimposing BMPR2 and BMPR2$^{\text{Mimic-ACVR2B}}$ to Activin A bound ACVR2B (PDB: 1S4Y) and docking, the Interface RMSD (IRMSD) was plotted against the score of the residues (ISC) in the docking interface. Datapoints of the final dockings (surface & cartoon representation, lower right corner of the plot) are highlighted in red. A structural comparison of the final docking vs. a simple superimposition is shown right next to the plot for both BMPR2 and BMPR2$^{\text{Mimic-ACVR2B}}$.

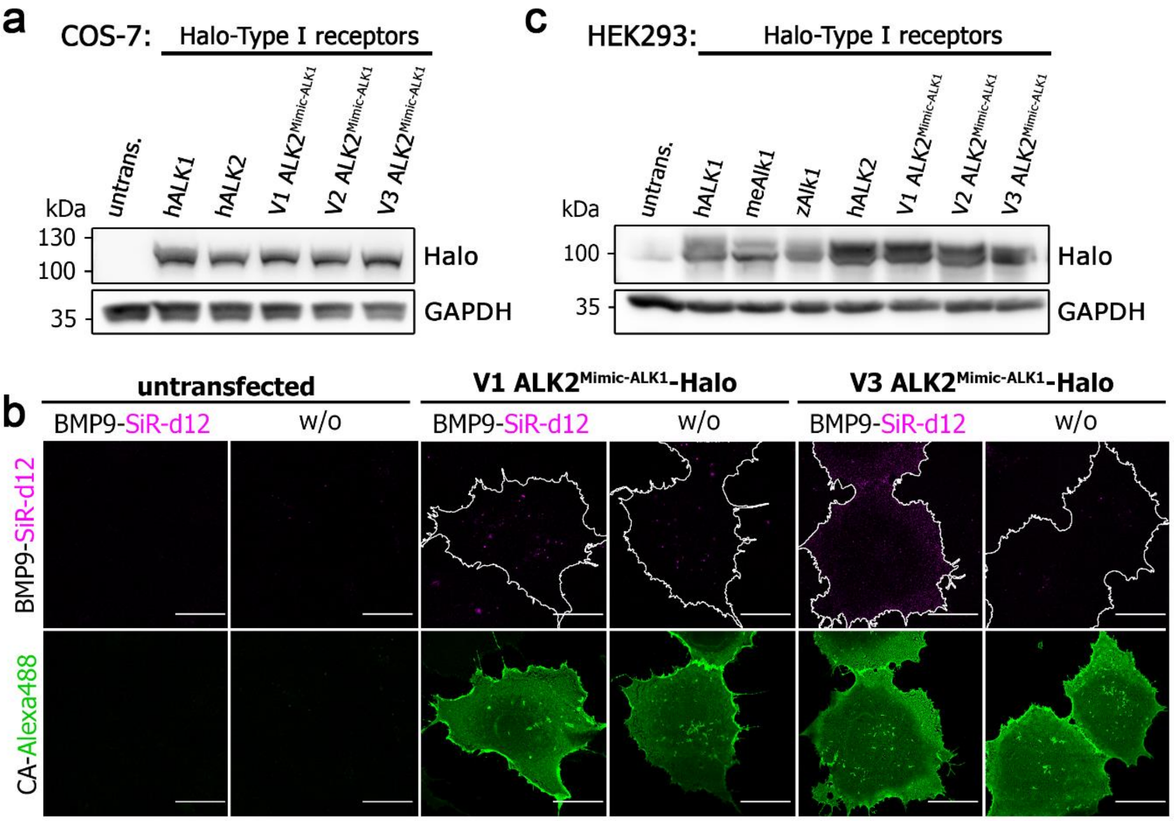

Supplementary Figure 12: ***Supplement to Fig.5,*** **a** Expression of Halo-tagged receptors ALK1-Halo, ALK2-Halo, V1 ALK2$^{Mimic\text{-}ALK1}$-Halo, V2 ALK2$^{Mimic\text{-}ALK1}$ and V3 ALK2$^{Mimic\text{-}ALK1}$-Halo in COS7- cells validated by Western blotting with a specific antibody directed against Halo-tag, using GAPDH as loading control. **b** Untransfected COS-7 cells or cells transiently expressing V1 ALK2$^{Mimic\text{-}ALK1}$-Halo or V2 ALK2$^{Mimic\text{-}ALK1}$-Halo were 24 hours post transfection simultaneously incubated with Halo-tag substrate CA-Alexa488 (green) and Activin A-Cy5 (magenta). Representative confocal microscopy images of untransfected COS-7 cells incubated with CA-Alexa488 and simultaneously stimulated with Activin A-Cy5 or PBS as control. Scale bar ≙ 20 µm. **c** Expression of Halo-tagged receptors ALK1, meAlk1, zAlk1, ALK2-Halo, V1 ALK2$^{Mimic\text{-}ALK1}$-Halo, V2 ALK2$^{Mimic\text{-}ALK1}$ and V3 ALK2$^{Mimic\text{-}ALK1}$-Halo in HEK293 cells validated by Western blotting with a specific antibody directed against Halo-tag, using GAPDH as loading control.

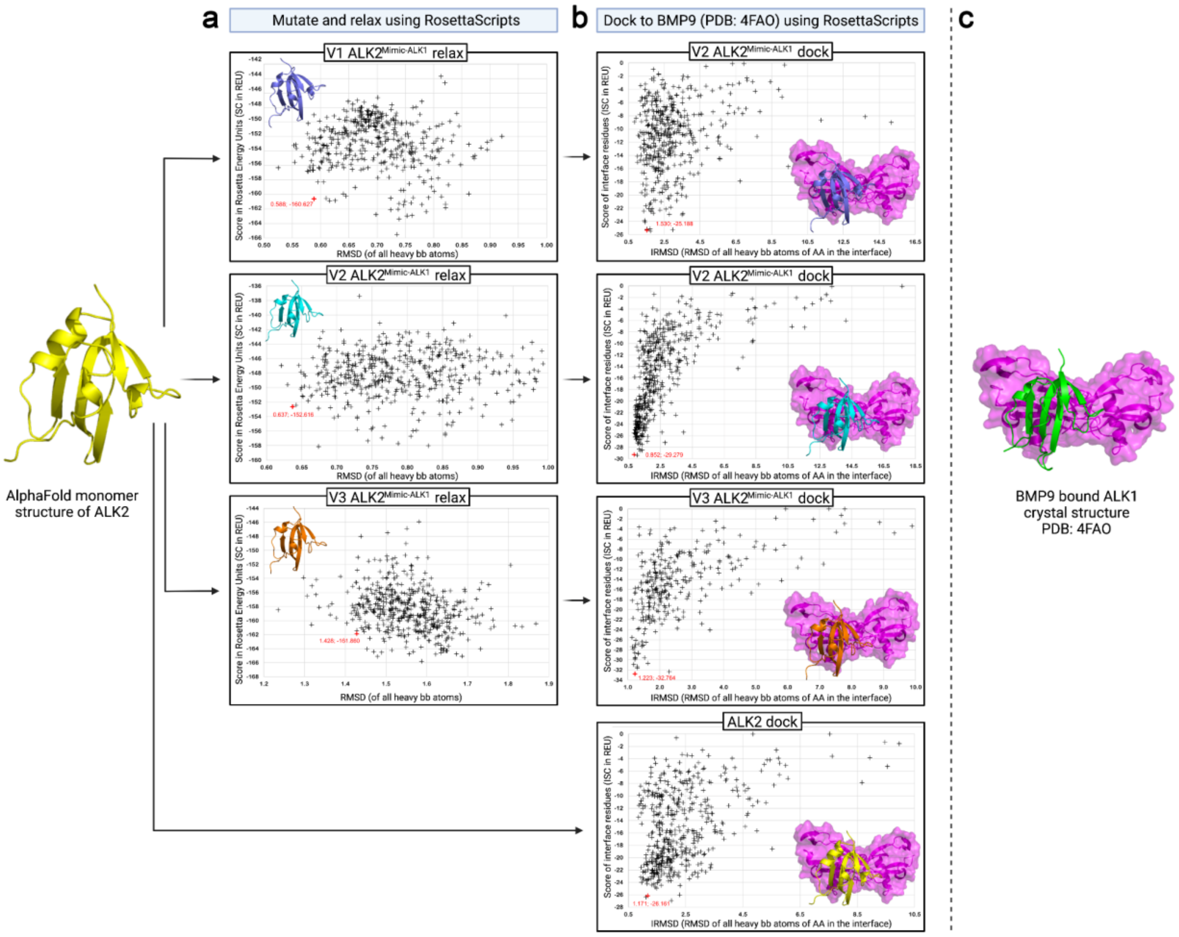

Supplementary Figure 13: ***Supplement to Fig.5*** **a** Simplified, schematic overview of the *in silico* homology modelling of the variants V1-, V2- and V3 $ALK2^{Mimic-ALK1}$ and docking of all variants and ALK2 to Activin A using the Rosetta Commons Modelling Suite. The basis for the modelling of the variants is the AlphaFold generated structure of ALK2. **a** RMSD of all heavy backbone atoms vs. the Rosetta-Score (scoring function ref2015), after introducing mutations and relaxing using the Rosetta Commons Modelling Suite, is plotted for 500 attempts for each variant. The Datapoint of the final homology model (cartoon rep. upper left corner of the plot) is highlighted in red. **b** After superimposing all variants and ALK2 to a BMP9 bound ALK1 (PDB: 4FAO) crystal structure and docking using Rosetta, the Interface RMSD (IRMSD) was plotted against the score of the docking interface residues (ISC). Datapoints of the final docked homology models (surface & cartoon representation, lower right corner of the plot) are highlighted in red. **c** Cartoon and surface representation of the crystal structure PDB: 4FAO of ALK1 bound to BMP9.

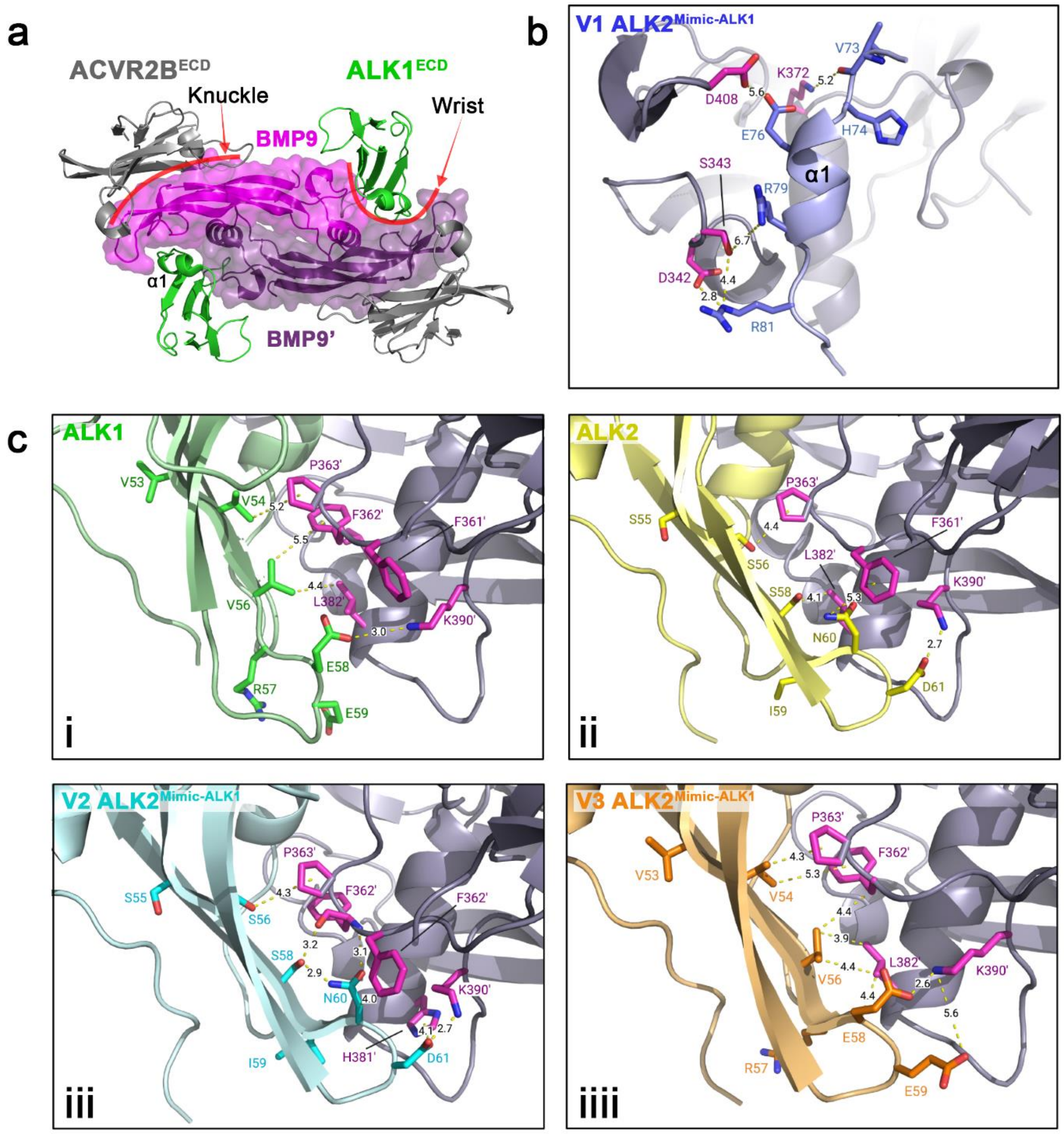

Supplementary Figure 14: ***Supplement to Fig.5,*** **a** Top view of the extracellular domains of ALK1 and ACVR2B in a heterotetrameric receptor complex bound to BMP9 (crystal structure PDB: 4FAO). Type I receptor ALK1 binds at the wrist interface of BMP9. **b** "HERR"-motif residues of V1 ALK2$^{Mimic-ALK1}$ in cartoon and stick representation. Distance measurements in Angstrom between V1 ALK2$^{Mimic-ALK1}$ and BMP9 were performed in PyMOL. **c** Residues that were mutated to generate the variant V3 ALK2$^{Mimic-ALK1}$ from V2 ALK2$^{Mimic-ALK1}$ are shown in stick representation in ALK1 (i), ALK2 (ii), V2 ALK2$^{Mimic-ALK1}$ (iii) and V3 ALK2$^{Mimic-ALK1}$ (iiii).

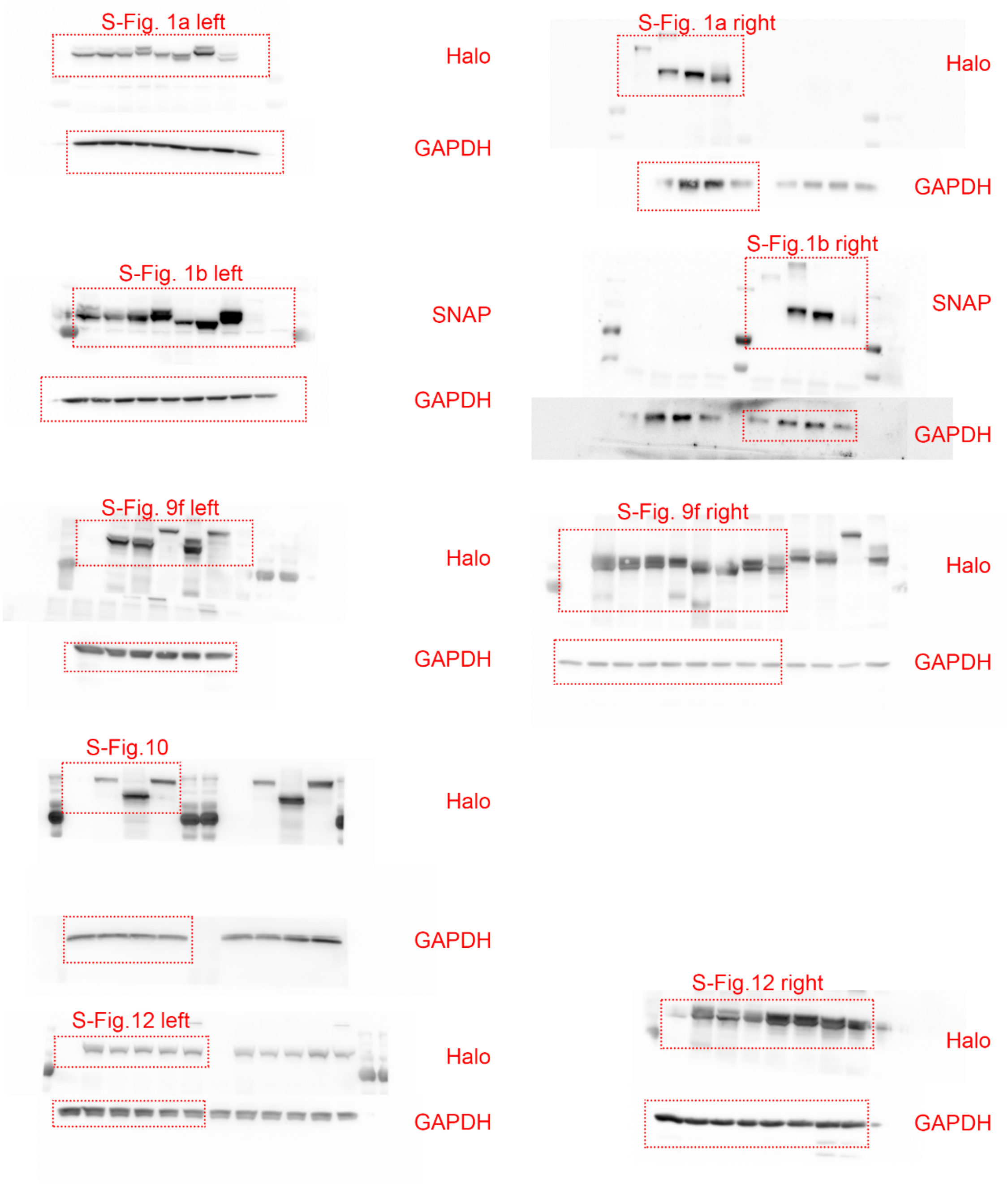

Supplementary Figure 15: ***Uncropped and unedited blot/gel images.***

Table S1. Oligonucleotides used for cloning.

Name & Application	Oligonucleotide-sequence (5'-3')
**Halo & SNAP-tagged TGFβ receptor family cloning**	
SNAP BamHI for	GCTGCT**GGATCC**GACAAAGACTGCGAAATGAAG
SNAP HindIII for	CTAGCA**AAGCTT**GACAAAGACTGCGAAATGAAG
SNAP Gly5 EcoRI rev	ACGCC**GAATTC**TCCACCTCCACCTCCACCCAGCCCAGGCTTGCCCAGTC
HALO BamHI for	GCTGCT**GGATCC**GCAGAAATCGGTACTGGCTTTCC
HALO HindIII for	CTAGCA**AAGCTT**GCAGAAATCGGTACTGGCTTTCC
HALO Gly5 EcoRI rev	AACGCC**GAATTC**TCCACCTCCACCTCCGCCGGAAATCTCGAGCGTCG
hALK1 EcoRI for	GGTGGA**GAATTC**GACCCTGTGAAGCCGTCTCGGGGCCCGCTG
hALK1 NotI rev	CCACCT**GCGGCCGC**ACCCTCGAGTTGAATCACTTTAGGC
hALK2 EcoRI for	GGTGGA**GAATTC**ATGGAAGATGAGAAGCCCAAGGTCAACC
hALK2 NotI rev	CCACCT**GCGGCCGC**ACAGTCAGTTTTCAATTTGTCGAG
hALK3 EcoRI for	GGTGGA**GAATT**CCAGAATCTGGATAGTATGCTTCATG
hALK3 NotI rev	CCACCT**GCGGCCGC**GATTTTTACATCTTGGGATTCAAC
hALK4 EcoRI for	GGTGGA**GAATTC**TCCGGGCCCCGGGGGGTCCAG
hALK4 NotI rev	CCACCT**GCGGCCGC**GATCTTCACGTCTTCCTGCAC
hALK5 EcoRI for	GGTGGA**GAATTC**CTGCTCCCGGGGGCGACGGCGTTAC
hALK5 NotI rev	CCACCT**GCGGCCGC**ACCCTCGAGCATTTTGATGCCTTCC
hALK6 EcoRI for	GGTGGA**GAATT**CAAGAAGGAGGATGGAGAGAGTACAGC
hALK6 NotI rev	CCACCT**GCGGCCGC**GAGTTTAATGTCCTGGGACTCTGAC
rALK7 EcoRI for	GGTGGA**GAATTC**CTGAAGTGTGTGTGTCTTTTG
rALK7 NotI rev	CCACCT**GCGGCCGC**GGCCTTACAGTCTTCCTTGAC
hACVR2A EcoRI for	GGTGGA**GAATT**CATGGGAGCTGCTGCAAAGTTGG
hACVR2A NotI rev	GCTCGA**GCGGCCGC**CTATAGACTAGATTCTTTGGG
hACVR2B EcoRI for	GGTGGA**GAATT**CGAGGCTGAGACCCGGGAGTGC
hACVR2B NotI rev	GCTCGA**GCGGCCGC**CTAGATGCTGGACTCTTTAGG
hTGFBR2 EcoRI for	GGTGGA**GAATTC**ATCCCACCGCACGTTCAGAAGTCG
hTGFBR2 NotI for	GCTCGA**GCGGCCGC**CTATTTGGTAGTGTTTAGGGAG
**BMPR2-Mimic-ACVR2B and ALK2-Mimic-ALK1 mutagenesis**	
BMPR2-Mimic-ACVR2B for	CTGGACGACTTCAACGAGTGTCACTATGAAGAATG
BMPR2-Mimic-ACVR2B rev	CCAACATCCTTGTTTTACAAGATTTATG
V1 ALK2-Mimic-ALK1 for	TGTAGAACCAGACCGTCCCCTGGCCAAGCTGTG
V1 ALK2-Mimic-ALK1 rev	GGTCTCCTTGTGAACCTGGAAGCAGCCTTTCTGG
V2 ALK2-Mimic-ALK1 for	AGAGGCAGACCGACCGAGTTCGTGGCTGTGGAGTGCTGCCAAGG
V2 ALK2-Mimic-ALK1 rev	ACACAGCTCCTTGTGCAGGTTGAAGCAGCCTTTGTGGTAGACGTGG
V3 ALK2-Mimic-ALK1 for	GAGGAGGGCTTCCACGTCTACCACAAAGGCTGCTTC
V3 ALK2-Mimic-ALK1 rev	TCTCACCAGCACCACAAAGCACTGCTGGCCTTCACAG
**Teleost: Halo- & SNAP-tagge medaka Alk1 & zebrafish Alk1 cloning**	
**mALK1-EcoR1-for**	GGTGGA**GAATT**CGACAGCAACGATGATGCAAATGACAGG
mALK1- NotI-rev	CCACCT**GCGGCCGC**GATGTCCTGCTTGACTTTGCTGAG
**zALK1-EcoR1-for**	GGTGGA**GAATT**CACAACTGCAGATCATCTTATGG
zALK1-Not1-rev	CCACCT**GCGGCCGC**GAGGTCCAGTTTAAGCTTGTC

Synthesis

2-(7-(Bis(methyl-*d*3)amino)-3-(bis(methyl-*d*3)iminio)-5,5-dimethyl-3,5-dihydrodibenzo[*b*,*e*]silin-10-yl)-4-(((2,5-dioxopyrrolidin-1-yl)oxy)carbonyl)benzoate (**NHS-SiR-d12**)

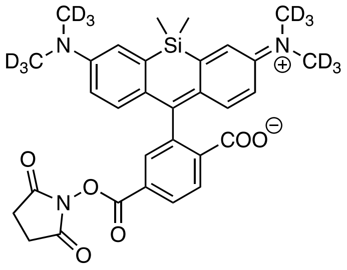

A 4 mL vial was charged with SiR-d12-6-COOH[1] (300 µg, 625 nmol, 1.0 equiv.), dissolved in DMF (200 µL) and DIPEA (0.33 µL, 1.88 µmol, 3.0 equiv.) before TSTU (226 µg, 750 nmol, 1.2 equiv.) was added in one portion from a stock solution in DMF. The reaction mixture was allowed to incubate for 1 h before it was quenched by addition of HOAc (0.18 µL, 3.13 µmol, 5.0 equiv.), diluted with 800 µL of a MeCN/H_2O mixture (50:50) and subjected to RP-HPLC purification. The product containing fractions were pooled and lyophilized to obtain SiR-d12-NHS (364 µg, 611 nmol, 98%) as a blue powder.

NOTE: Preparing aliquots for single use is recommended that can be stored at –20 °C.

LRMS (ESI): calc. for $C_{31}H_{20}D_{12}N_3O_6Si^+$ $(M+H)^+$: 582.3; found: 582.3

References:

1 Liu, B. *et al.* Fatty acid binding proteins shape the cellular response to activation of the glucocorticoid receptor. *bioRxiv* (2021).

2 Hao, Q. *et al.* The S-phase-induced lncRNA SUNO1 promotes cell proliferation by controlling YAP1/Hippo signaling pathway. *Elife* **9**, doi:10.7554/eLife.55102 (2020).

3 Goebel, E. J. *et al.* Structures of activin ligand traps using natural sets of type I and type II TGFbeta receptors. *iScience* **25**, 103590, doi:10.1016/j.isci.2021.103590 (2022).

4 Townson, S. A. *et al.* Specificity and structure of a high affinity activin receptor-like kinase 1 (ALK1) signaling complex. *J Biol Chem* **287**, 27313-27325, doi:10.1074/jbc.M112.377960 (2012).

5 Radaev, S. *et al.* Ternary complex of transforming growth factor-beta1 reveals isoform-specific ligand recognition and receptor recruitment in the superfamily. *J Biol Chem* **285**, 14806-14814, doi:10.1074/jbc.M109.079921 (2010).

6 Liu, Y. *et al.* The Cation-pi Interaction Enables a Halo-Tag Fluorogenic Probe for Fast No-Wash Live Cell Imaging and Gel-Free Protein Quantification. *Biochemistry* **56**, 1585-1595, doi:10.1021/acs.biochem.7b00056 (2017).
