# Supplementary material for: A versatile Halo- and SNAP-tagged BMP/TGFβ receptor library for quantification of cell surface ligand binding
Source: Commun Biol. 2023 Jan 12;6:34. doi: 10.1038/s42003-022-04388-4 (PMC9837045; doi:10.1038/s42003-022-04388-4)
Supplement: Supplementary file 3 — Description of Additional Supplementary Files [file 42003_2022_4388_MOESM3_ESM.pdf]

Description of Additional Supplementary Files

File name: Supplementary Movie 1

Description: : Live Cell Imaging Movie of ActA-Cy5 binding on ACVR2B-Halo transfected COS-7 cells

File name: Supplementary Data 1

Description: Numerical source data for graphs in main and supplemental figures
