# Supplementary material for: A versatile Halo- and SNAP-tagged BMP/TGFβ receptor library for quantification of cell surface ligand binding
Source: Commun Biol. 2023 Jan 12;6:34. doi: 10.1038/s42003-022-04388-4 (PMC9837045; doi:10.1038/s42003-022-04388-4)
Supplement: Supplementary file 6 — Reporting Summary [file 42003_2022_4388_MOESM6_ESM.pdf]

nature portfolio

Corresponding author(s): Petra Knaus

Last updated by author(s): Oct 5, 2022

Reporting Summary

Nature Portfolio wishes to improve the reproducibility of the work that we publish. This form provides structure for consistency and transparency in reporting. For further information on Nature Portfolio policies, see our Editorial Policies and the Editorial Policy Checklist.

Statistics

For all statistical analyses, confirm that the following items are present in the figure legend, table legend, main text, or Methods section.

n/a	Confirmed	
☐	☒	The exact sample size (*n*) for each experimental group/condition, given as a discrete number and unit of measurement
☐	☒	A statement on whether measurements were taken from distinct samples or whether the same sample was measured repeatedly
☐	☒	The statistical test(s) used AND whether they are one- or two-sided<br>*Only common tests should be described solely by name; describe more complex techniques in the Methods section.*
☒	☐	A description of all covariates tested
☐	☒	A description of any assumptions or corrections, such as tests of normality and adjustment for multiple comparisons
☐	☒	A full description of the statistical parameters including central tendency (e.g. means) or other basic estimates (e.g. regression coefficient) AND variation (e.g. standard deviation) or associated estimates of uncertainty (e.g. confidence intervals)
☒	☐	For null hypothesis testing, the test statistic (e.g. *F*, *t*, *r*) with confidence intervals, effect sizes, degrees of freedom and *P* value noted<br>*Give P values as exact values whenever suitable.*
☒	☐	For Bayesian analysis, information on the choice of priors and Markov chain Monte Carlo settings
☒	☐	For hierarchical and complex designs, identification of the appropriate level for tests and full reporting of outcomes
☒	☐	Estimates of effect sizes (e.g. Cohen's *d*, Pearson's *r*), indicating how they were calculated

Our web collection on statistics for biologists contains articles on many of the points above.

Software and code

Policy information about availability of computer code

Data collection	Abberior Imspector Acquisition & Analysis Software v16.3, TECAN i-control V3.7
Data analysis	GraphPad Prism 9.3, FIJI, PyMOL, All used scripts can be found in an open-source repository under https://github.com/Habacef/Supplementary-scripts-for-LSBA.

For manuscripts utilizing custom algorithms or software that are central to the research but not yet described in published literature, software must be made available to editors and reviewers. We strongly encourage code deposition in a community repository (e.g. GitHub). See the Nature Portfolio guidelines for submitting code & software for further information.

Data

Policy information about availability of data

All manuscripts must include a data availability statement. This statement should provide the following information, where applicable:

- Accession codes, unique identifiers, or web links for publicly available datasets
- A description of any restrictions on data availability
- For clinical datasets or third party data, please ensure that the statement adheres to our policy

The data supporting the findings of this study are available from the corresponding author upon request.

March 2021

Human research participants

Policy information about studies involving human research participants and Sex and Gender in Research.

Reporting on sex and gender	na
Population characteristics	na
Recruitment	na
Ethics oversight	na

Note that full information on the approval of the study protocol must also be provided in the manuscript.

Field-specific reporting

Please select the one below that is the best fit for your research. If you are not sure, read the appropriate sections before making your selection.

☒ Life sciences ☐ Behavioural & social sciences ☐ Ecological, evolutionary & environmental sciences

For a reference copy of the document with all sections, see nature.com/documents/nr-reporting-summary-flat.pdf

Life sciences study design

All studies must disclose on these points even when the disclosure is negative.

Sample size	In order to account for the variablity of transient receptor expression at least 10 images were taken per condition, allowing a correlation analysis of ligand binding vs. receptor expression. As a positive correlation was detected for known ligand/receptor pairs such as TGFβ1/ TGFBR2 or BMP9/ALK1, 10 images per condition were considered high enough to cover any occuring variation in receptor expression.
Data exclusions	Data points representing high LSBA values compared to the mean of the condition and which originated from imaging artefacts were excluded from the analysis.
Replication	Experiments were repeated at least three times individually. BMP9, ActivinA and TGFβ1 LSBA data was collected by two independent investigators. All attempts of replication were successful.
Randomization	Randomization was not relevant to our study as data analysis was semi-automated using the described FIJI scripts.
Blinding	Blinding was not possible as the investigators performed the experiment and recorded the data using the microscope. Data anaylsis however was semi-automated using the FIJI script described in the method section.

Reporting for specific materials, systems and methods

We require information from authors about some types of materials, experimental systems and methods used in many studies. Here, indicate whether each material, system or method listed is relevant to your study. If you are not sure if a list item applies to your research, read the appropriate section before selecting a response.

Materials & experimental systems

n/a	Involved in the study	
☐	☒	Antibodies
☐	☒	Eukaryotic cell lines
☒	☐	Palaeontology and archaeology
☒	☐	Animals and other organisms
☒	☐	Clinical data
☒	☐	Dual use research of concern

Methods

n/a	Involved in the study	
☒	☐	ChIP-seq
☐	☒	Flow cytometry
☒	☐	MRI-based neuroimaging

Antibodies

Antibodies used	anti-GAPDH (Cell Signaling; #2118; monoclonal rabbit antibody), anti-Halo (ProMega; #G9211; monoclonal mouse antibody) anti-SNAP (Invitrogen; #CAB4255; polyclonal rabbit antibody) and goat-α-mouse or goat-α-rabbit IgG HRP conjugates (± 0.8 mg/ml, Dianova; #111-035-144, #115-035-068)

Validation	Halo and SNAP antibody specificity was confirmed by transient expression of Halo- or SNAP-tagged BMPRs without any detection of crossreactivity.

Eukaryotic cell lines

Policy information about cell lines and Sex and Gender in Research

Cell line source(s)	COS-7 cells and HEK293t were obtained from the German Collection of Microorganisms and Cell Cultures (DSMZ)
Authentication	None of the cell lines used were authenticated.
Mycoplasma contamination	All cell lines tested negative for mycoplasma contamination.
Commonly misidentified lines (See ICLAC register)	No commonly misidentified lines were used.

Flow Cytometry

Plots

Confirm that:

☒ The axis labels state the marker and fluorochrome used (e.g. CD4-FITC).

☒ The axis scales are clearly visible. Include numbers along axes only for bottom left plot of group (a 'group' is an analysis of identical markers).

☐ All plots are contour plots with outliers or pseudocolor plots.

☒ A numerical value for number of cells or percentage (with statistics) is provided.

Methodology

Sample preparation	For FACS analysis of ligand binding to COS-7 cells transiently expressing Halo-receptor fusion proteins, 600,000 cells were transfected in 6 wells with desired constructs. 24 hours post-transfection, cells were placed on ice, and washed twice with ice-cold DPBS followed by an incubation with fluorescent Halo-ligand CA-Alexa488 (0.5 µM) in the absence or presence of Activin A-Cy5 (2 nM) for 30 minutes at 4 °C. Subsequently, cells were washed once with ice-cold DPBS before incubation with Accutase solution at RT for 15 min. After detachment, cells were resuspended in DPBS and centrifuged at (1000 n/min) for 5 mins. Finally, cells were resuspended in PBS (100 mM EDTA) and measured in BD LSRFortessa™ Cell Analyzer.
Instrument	BD LSRFortessa™ Cell Analyzer
Software	BD FACSDiva 8.0.1 software
Cell population abundance	1000 transfected cells with a high signal in the FITC channel were present in all samples
Gating strategy	Cells were gated first for single cells using SSC-A/FSC-A, followed by a gating of 1000 transfected cells with a high signal in the FITC channel. Activin A-Cy5 surface binding was quantified as mean Cy5 intensity of AF488+ cells.

☒ Tick this box to confirm that a figure exemplifying the gating strategy is provided in the Supplementary Information.
